# Correction: The novel microRNAs hsa-miR-nov7 and hsa-miR-nov3 are over-expressed in locally advanced breast cancer

**DOI:** 10.1371/journal.pone.0253361

**Published:** 2021-06-10

**Authors:** Deepak Poduval, Zuzana Sichmanova, Anne Hege Straume, Per Eystein Lønning, Stian Knappskog

Fig 1 is missing part B. The authors have provided a corrected version here.

Fig 8 is missing part B. The authors have provided a corrected version here.

**Fig 1:**
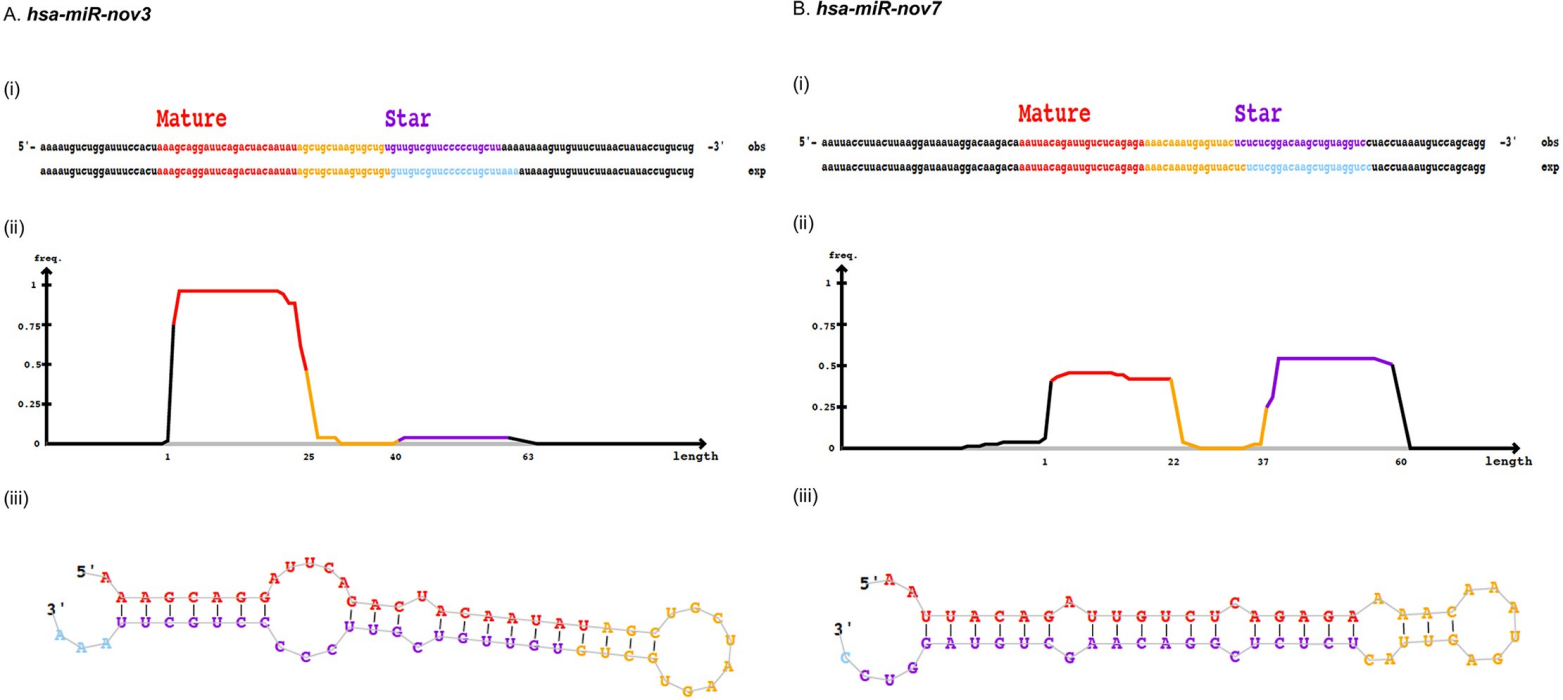
Predicted novel miRNAs. Depiction of novel miRNAs (A) *hsa-miR-nov3* and (B) *hsa-miR-nov7*, identified by miRDeep2, showing (i) predicted mature and star sequences, **exp**, probabilistic model expected from Drosha/Dicer processing and **obs**, observed sequences from sequencing data (ii) density plot for read counts for mature and star sequences as well as (iii) miRNA secondary structure.

**Fig 8:**
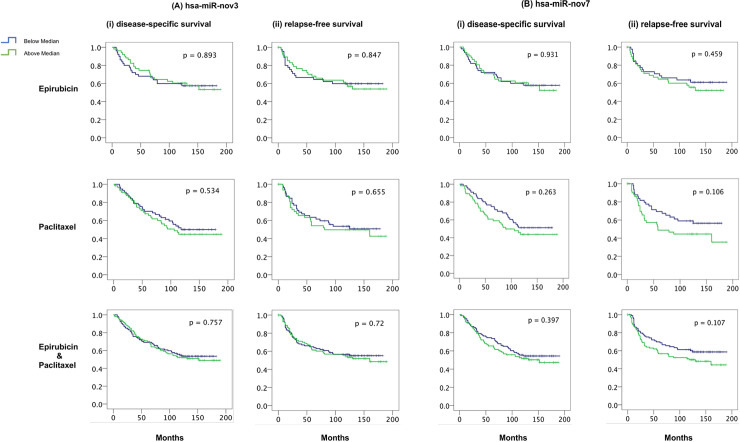
miRNAs and breast cancer survival. Kaplan-Meier curves showing (i) disease-specific and (ii) relapse-free survival of locally advanced breast cancer patients treated with epirubicin or paclitaxel monotherapy in the neoadjuvant setting (study 1), with respect to expression levels of (A) *hsa-miR-nov3* and (B) *hsa-miR-nov7* on all samples.
