# Supplementary material for: The novel microRNAs hsa-miR-nov7 and hsa-miR-nov3 are over-expressed in locally advanced breast cancer
Source: PLoS One. 2020 Apr 16;15(4):e0225357. doi: 10.1371/journal.pone.0225357 (PMC7162276; doi:10.1371/journal.pone.0225357)
Supplement: S1 Fig — Table on the upper left shows miRDeep2 scores and read counts. RNA secondary structure for miRNA on the top right. Color code for depiction as follows mature sequence in red, loop sequence in yellow and purple for star sequences. Density plot in the middle shows distribution of reads in precursor reads predicted. Dotted lines illustrate alignment and mm, number of mismatches. Exp, is potential precursor model predicted by algorithm with taking accounts of stability based on free energy, position and read frequencies according to Dicer/Drosha processing of miRNA. Obs, is position and reads found from deep sequencing data. (A) hsa-miR-nov3 and (B) hsa-miR-nov7. (DOCX) [file pone.0225357.s001.docx]

A)


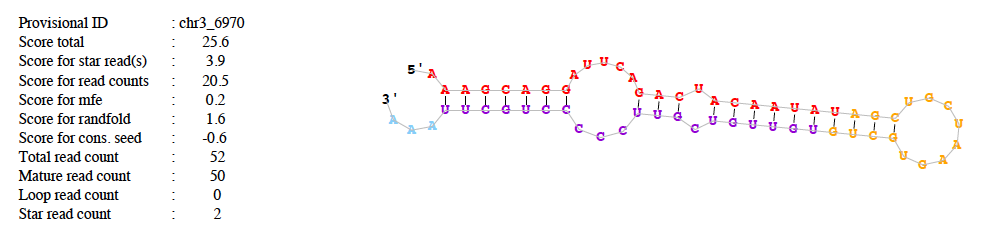


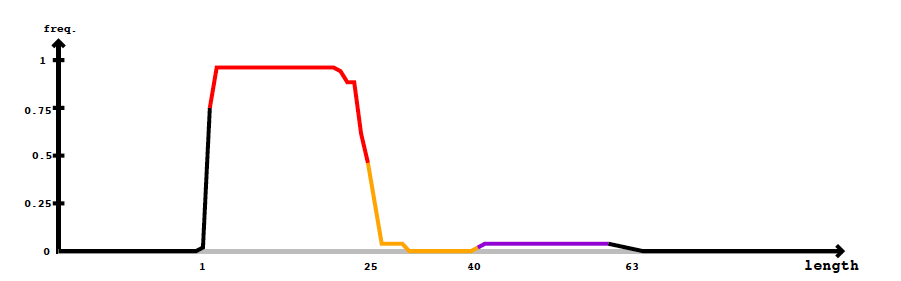


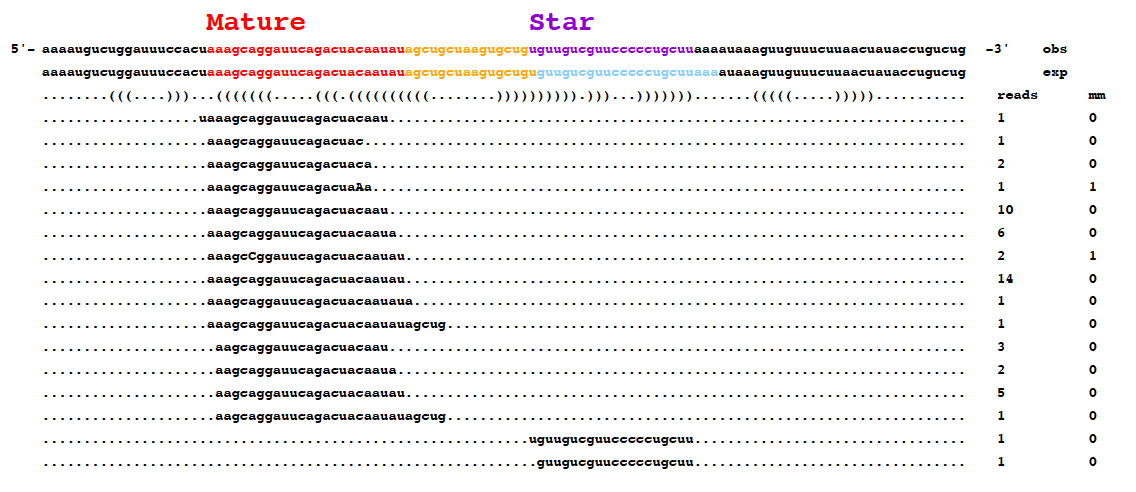


B)


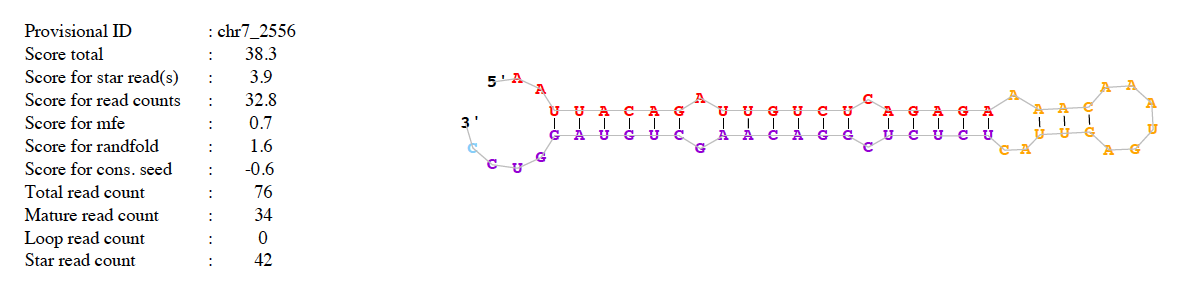


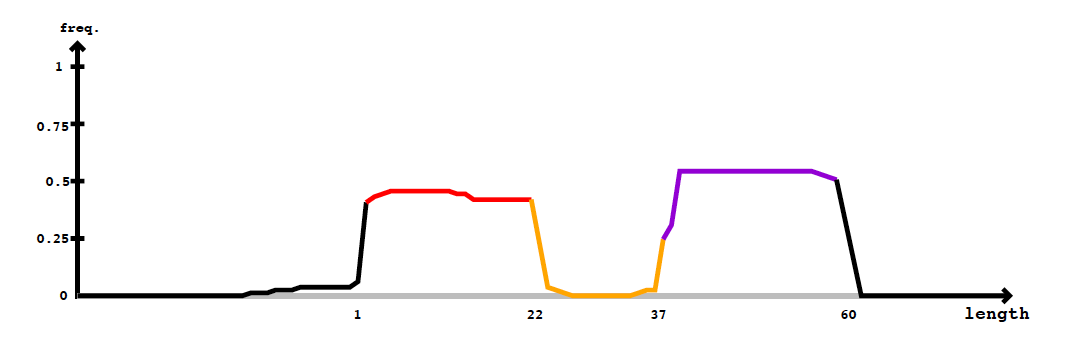


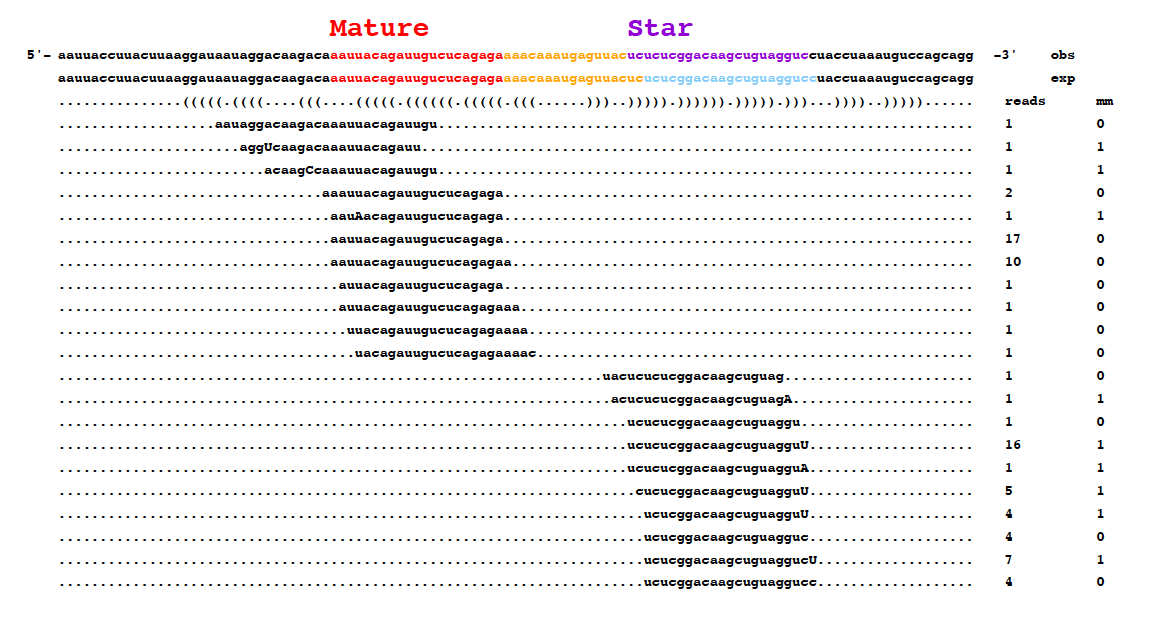


**Figure 1**: Predicted novel miRNAs. Table on the upper left shows miRDeep2 scores and read counts. RNA secondary structure for miRNA on the top right. Color code for depiction as follows mature sequence in red, loop sequence in yellow and purple for star sequences. Density plot in the middle shows distribution of reads in precursor reads predicted. Dotted lines illustrate alignment and mm, number of mismatches. Exp, is potential precursor model predicted by algorithm with taking accounts of stability based on free energy, position and read frequencies according to Dicer/Drosha processing of miRNA. Obs, is postion and reads found from deep sequencing data. (A) *hsa-miR-nov3* and (B) *hsa-miR-nov7*.
