# Supplementary material for: The novel microRNAs hsa-miR-nov7 and hsa-miR-nov3 are over-expressed in locally advanced breast cancer
Source: PLoS One. 2020 Apr 16;15(4):e0225357. doi: 10.1371/journal.pone.0225357 (PMC7162276; doi:10.1371/journal.pone.0225357)
Supplement: S1 Table — Panel generated based on CGPv2/3-panels [41], Roche’s Comprehensive Cancer Design along with manual literature search, to filter target genes of interest. (DOCX) [file pone.0225357.s002.docx]

**Supporting Table S1**. In-house pan-cancer panel of 283 tumor suppressor genes, generated based on CGPv2/3-panels[1], Roche’s Comprehensive Cancer Design along with manual literature search, to filter target genes of interest.

| **Gene Name** | **Chromosome** |
| --- | --- |
| AIP | chr11 |
| ALDH2 | chr12 |
| AMER1 | chrX |
| APC | chr5 |
| AR | chrX |
| ARHGAP26 | chr5 |
| ARHGEF12 | chr11 |
| ARID1A | chr1 |
| ARID1B | chr6 |
| ARID2 | chr12 |
| ARID4A | chr14 |
| ASXL1 | chr20 |
| ATM | chr11 |
| ATR | chr3 |
| ATRX | chrX |
| AXIN2 | chr17 |
| BAP1 | chr3 |
| BARD1 | chr2 |
| BCL7A | chr12 |
| BLID | chr11 |
| BLM | chr15 |
| BMP2 | chr20 |
| BMP3 | chr4 |
| BMP4 | chr14 |
| BMP7 | chr20 |
| BMPR1A | chr10 |
| BRCA1 | chr17 |
| BRCA2 | chr13 |
| BRIP1 | chr17 |
| BTG1 | chr12 |
| BUB1B | chr15 |
| CARS | chr11 |
| CASC5 | chr15 |
| CASP8 | chr2 |
| CCDC6 | chr10 |
| CCNB1IP1 | chr14 |
| CD2 | chr1 |
| CDC73 | chr1 |
| CDH1 | chr16 |
| CDH11 | chr16 |
| CDH13 | chr16 |
| CDK12 | chr17 |
| CDK2AP2 | chr11 |
| CDKN1A | chr6 |
| CDKN1B | chr12 |
| CDKN1C | chr11 |
| CDKN2A | chr9 |
| CDKN2B | chr9 |
| CDKN2C | chr1 |
| CDKN2D | chr19 |
| CDX2 | chr13 |
| CEBPA | chr19 |
| CHD5 | chr1 |
| CHD6 | chr20 |
| CHEK1 | chr11 |
| CHEK2 | chr22 |
| CHFR | chr12 |
| CHN1 | chr2 |
| CIC | chr19 |
| CIITA | chr16 |
| CLDN3 | chr7 |
| CLDN4 | chr7 |
| CLTCL1 | chr22 |
| CNBP | chr3 |
| COX6C | chr8 |
| CREB3L1 | chr11 |
| CREBBP | chr16 |
| CTCFL | chr20 |
| CTNNB1 | chr3 |
| CYLD | chr16 |
| DAPK1 | chr9 |
| DDB2 | chr11 |
| DDIT3 | chr12 |
| DDX53 | chrX |
| DICER1 | chr14 |
| DKK1 | chr10 |
| DNMT3A | chr2 |
| EBF1 | chr5 |
| EIF4A2 | chr3 |
| ELAC2 | chr17 |
| EMP3 | chr19 |
| EP300 | chr22 |
| EPHA5 | chr4 |
| EPHA6 | chr3 |
| EPHB6 | chr7 |
| ERCC1 | chr19 |
| ERCC2 | chr19 |
| ERCC3 | chr2 |
| ERCC4 | chr16 |
| ERCC5 | chr13 |
| ERG | chr21 |
| ESR1 | chr6 |
| EXT1 | chr8 |
| EXT2 | chr11 |
| FAM46C | chr1 |
| FANCA | chr16 |
| FANCB | chrX |
| FANCC | chr9 |
| FANCD2 | chr3 |
| FANCE | chr6 |
| FANCF | chr11 |
| FANCG | chr9 |
| FANCI | chr15 |
| FANCL | chr2 |
| FANCM | chr14 |
| FAS | chr10 |
| FAT1 | chr4 |
| FBXO11 | chr2 |
| FBXW7 | chr4 |
| FH | chr1 |
| FHIT | chr3 |
| FHL1 | chrX |
| FLCN | chr17 |
| FOXL2 | chr3 |
| FOXO1 | chr13 |
| FOXO3 | chr6 |
| FOXO4 | chrX |
| FUS | chr16 |
| GAS7 | chr17 |
| GATA1 | chrX |
| GATA2 | chr3 |
| GATA3 | chr10 |
| GATA4 | chr8 |
| GATA5 | chr20 |
| GMPS | chr3 |
| GPC3 | chrX |
| GSTM1 | chr1 |
| GSTP1 | chr11 |
| HAND2 | chr4 |
| HECW1 | chr7 |
| HERPUD1 | chr16 |
| HIC1 | chr17 |
| HNF1A | chr12 |
| HOXA10 | chr7 |
| HOXA11 | chr7 |
| HOXA9 | chr7 |
| ID4 | chr6 |
| IGFBP3 | chr7 |
| IKZF1 | chr7 |
| IL21R | chr16 |
| KDM5C | chrX |
| KDM6A | chrX |
| KDSR | chr18 |
| KEAP1 | chr19 |
| KL | chr13 |
| KLF6 | chr10 |
| KMT2C | chr7 |
| KMT2D | chr12 |
| LMNA | chr1 |
| LRP5 | chr11 |
| LTBP2 | chr14 |
| MAL | chr2 |
| MC1R | chr16 |
| MEN1 | chr11 |
| MGMT | chr10 |
| MIR124-1 | chr8 |
| MIR127 | chr14 |
| MIR155 | chr21 |
| MLF1 | chr3 |
| MLH1 | chr3 |
| MLLT11 | chr1 |
| MNX1 | chr7 |
| MRE11A | chr11 |
| MSH2 | chr2 |
| MSH6 | chr2 |
| MTUS2 | chr13 |
| MUTYH | chr1 |
| NBN | chr8 |
| NCKIPSD | chr3 |
| NDRG1 | chr8 |
| NF1 | chr17 |
| NF2 | chr22 |
| NFKB2 | chr10 |
| NTRK3 | chr15 |
| NUMA1 | chr11 |
| OPTN | chr10 |
| PALB2 | chr16 |
| PAX5 | chr9 |
| PBRM1 | chr3 |
| PDCD1LG2 | chr9 |
| PER1 | chr17 |
| PGR | chr11 |
| PHF6 | chrX |
| PLAG1 | chr8 |
| PML | chr15 |
| PMS1 | chr2 |
| PMS2 | chr7 |
| PRDM1 | chr6 |
| PRDM16 | chr1 |
| PRDM2 | chr1 |
| PREX2 | chr8 |
| PRKAR1A | chr17 |
| PRKDC | chr8 |
| PRLR | chr5 |
| PTCH1 | chr9 |
| PTEN | chr10 |
| PTGS2 | chr1 |
| PTPN6 | chr12 |
| PTPRD | chr9 |
| PYCARD | chr16 |
| RAB40AL | chrX |
| RABEP1 | chr17 |
| RAD51B | chr14 |
| RAD51C | chr17 |
| RAD51D | chr17 |
| RANBP17 | chr5 |
| RAP1GDS1 | chr4 |
| RASSF1 | chr3 |
| RASSF5 | chr1 |
| RB1 | chr13 |
| RBBP8 | chr18 |
| RBM15 | chr1 |
| RBP1 | chr3 |
| RHOH | chr4 |
| RMI2 | chr16 |
| RNASEL | chr1 |
| RPTOR | chr17 |
| RRM1 | chr11 |
| RUNX1 | chr21 |
| RUNX1T1 | chr8 |
| RUNX3 | chr1 |
| SARDH | chr9 |
| SBDS | chr7 |
| SDHAF2 | chr11 |
| SDHB | chr1 |
| SDHC | chr1 |
| SDHD | chr11 |
| SETD2 | chr3 |
| SFPQ | chr1 |
| SFRP1 | chr8 |
| SFRP2 | chr4 |
| SFRP5 | chr10 |
| SLC5A8 | chr12 |
| SLX4 | chr16 |
| SMAD2 | chr18 |
| SMAD3 | chr15 |
| SMAD4 | chr18 |
| SMARCA4 | chr19 |
| SMARCB1 | chr22 |
| SNCG | chr10 |
| SOCS1 | chr16 |
| SOCS3 | chr17 |
| SPECC1 | chr17 |
| SPEN | chr1 |
| SRGAP3 | chr3 |
| STK11 | chr19 |
| SUFU | chr10 |
| SYK | chr9 |
| TCEA1 | chr8 |
| TET1 | chr10 |
| TFAP2A | chr6 |
| TFG | chr3 |
| TGFBR2 | chr3 |
| THBS1 | chr15 |
| THRAP3 | chr1 |
| TIMP3 | chr22 |
| TLX3 | chr5 |
| TMEFF2 | chr2 |
| TMEM127 | chr2 |
| TNFAIP3 | chr6 |
| TOP2A | chr17 |
| TP53 | chr17 |
| TP63 | chr3 |
| TP73 | chr1 |
| TRIM33 | chr1 |
| TSC1 | chr9 |
| TSC2 | chr16 |
| TSHR | chr14 |
| TTL | chr2 |
| TUBB3 | chr16 |
| VDR | chr12 |
| VHL | chr3 |
| WIF1 | chr12 |
| WRN | chr8 |
| XPA | chr9 |
| XPC | chr3 |
| YWHAE | chr17 |
| ZBTB16 | chr11 |
| ZMYM2 | chr13 |
| ZNF331 | chr19 |
| ZNF668 | chr16 |
| ZRSR2 | chrX |
| sep.09 | chr17 |

1. Yates LR, Gerstung M, Knappskog S, Desmedt C, Gundem G, Van Loo P, et al. Subclonal diversification of primary breast cancer revealed by multiregion sequencing. Nat Med. 2015;21(7):751-9. Epub 2015/06/23. doi: 10.1038/nm.3886. PubMed PMID: 26099045; PubMed Central PMCID: PMCPMC4500826.
