# Supplementary material for: The novel microRNAs hsa-miR-nov7 and hsa-miR-nov3 are over-expressed in locally advanced breast cancer
Source: PLoS One. 2020 Apr 16;15(4):e0225357. doi: 10.1371/journal.pone.0225357 (PMC7162276; doi:10.1371/journal.pone.0225357)
Supplement: S3 Table — Spearman correlation table for hsa-miR-nov3 (A) and hsa-miR-nov7 (B) inversely correlated tumor suppressor genes. (DOCX) [file pone.0225357.s004.docx]

**Supporting Table S2**. Spearman correlation table for *hsa-miR-nov3* (A) and *hsa-miR-nov7* (B) inversely correlated tumor suppressor genes.

A)

| **Gene** | **Estimate** | **P value** | **Mean Expression** | |
| --- | --- | --- | --- | --- |
| ARHGAP26 | -0.0065 | 0.9262 | 14.4132 |  |
| ARID2 | -0.0573 | 0.4160 | 15.5138 |  |
| ARID4A | -0.0134 | 0.8493 | 14.6255 |  |
| ATRX | -0.0483 | 0.4923 | 13.9469 |  |
| BCL7A | -0.0926 | 0.1876 | 14.1301 |  |
| BLID | -0.0529 | 0.4521 | 13.9765 |  |
| BMP3 | -0.0124 | 0.8600 | 13.3724 |  |
| BMP7 | -0.0004 | 0.9954 | 14.3881 |  |
| BRIP1 | -0.0486 | 0.4899 | 13.8642 |  |
| CARS | -0.0213 | 0.7627 | 15.8107 |  |
| CDH11 | -0.0518 | 0.4618 | 17.7525 |  |
| CDKN1A | -0.0217 | 0.7581 | 15.6141 |  |
| CDKN1C | -0.0144 | 0.8379 | 16.0217 |  |
| CDKN2A | -0.0842 | 0.2309 | 14.3086 |  |
| CDKN2B | -0.0310 | 0.6595 | 15.0449 |  |
| CDX2 | -0.0527 | 0.4537 | 13.5056 |  |
| CHD5 | -0.0314 | 0.6555 | 13.7651 |  |
| CHD6 | -0.0826 | 0.2401 | 15.8235 |  |
| CHFR | -0.0805 | 0.2522 | 17.0289 |  |
| CHN1 | -0.0013 | 0.9850 | 14.8979 |  |
| CIITA | -0.0062 | 0.9298 | 14.5010 |  |
| CLTCL1 | -0.1132 | 0.1071 | 14.1098 |  |
| CNBP | -0.0703 | 0.3177 | 16.3166 |  |
| CTNNB1 | -0.1208 | 0.0853 | 15.9912 |  |
| DAPK1 | -0.1016 | 0.1480 | 15.6762 |  |
| DDB2 | -0.0454 | 0.5191 | 15.2302 |  |
| DDIT3 | -0.0077 | 0.9129 | 16.0630 |  |
| DDX53 | -0.0291 | 0.6794 | 13.5698 |  |
| DKK1 | -0.0004 | 0.9954 | 14.3922 |  |
| ELAC2 | -0.1488 | 0.0336 | 16.1477 |  |
| EMP3 | -0.0232 | 0.7418 | 17.4996 |  |
| EP300 | -0.0645 | 0.3596 | 16.1435 |  |
| EPHA6 | -0.0616 | 0.3815 | 13.3497 |  |
| ERCC1 | -0.0019 | 0.9788 | 15.8715 |  |
| ERCC3 | -0.1056 | 0.1326 | 15.5411 |  |
| ERCC4 | -0.0049 | 0.9449 | 13.9151 |  |
| EXT2 | -0.0937 | 0.1824 | 15.4237 |  |
| FANCF | -0.0183 | 0.7952 | 13.9110 |  |
| FANCI | -0.0072 | 0.9187 | 15.5721 |  |
| FANCL | -0.0878 | 0.2119 | 14.9136 |  |
| FAT1 | -0.0280 | 0.6909 | 17.3311 |  |
| FBXO11 | -0.0567 | 0.4205 | 16.6127 |  |
| FHIT | -0.0265 | 0.7071 | 14.5957 |  |
| FHL1 | -0.0015 | 0.9831 | 16.5500 |  |
| FLCN | -0.0163 | 0.8168 | 14.3452 |  |
| FOXL2 | -0.0424 | 0.5466 | 13.3440 |  |
| GATA2 | -0.0145 | 0.8364 | 14.8464 |  |
| GATA3 | -0.0301 | 0.6686 | 16.8666 |  |
| GATA4 | -0.0356 | 0.6128 | 13.4347 |  |
| GATA5 | -0.1094 | 0.1193 | 13.5412 |  |
| GSTM1 | -0.0315 | 0.6543 | 15.4934 |  |
| HAND2 | -0.0792 | 0.2600 | 13.8374 |  |
| HIC1 | -0.0022 | 0.9746 | 15.0303 |  |
| HOXA10 | -0.0662 | 0.3468 | 14.3840 |  |
| HOXA11 | -0.0274 | 0.6970 | 13.1974 |  |
| HOXA9 | -0.0077 | 0.9126 | 13.3157 |  |
| IGFBP3 | -0.0380 | 0.5898 | 17.3472 |  |
| KDM6A | -0.0797 | 0.2569 | 16.1162 |  |
| KDSR | -0.0348 | 0.6209 | 16.9388 |  |
| KMT2C | -0.0108 | 0.8783 | 14.4106 |  |
| LMNA | -0.0361 | 0.6079 | 17.5236 |  |
| MLH1 | -0.0409 | 0.5617 | 16.5589 |  |
| MLLT11 | -0.0638 | 0.3646 | 16.2646 |  |
| MSH2 | -0.0441 | 0.5311 | 14.7562 |  |
| MSH6 | -0.0715 | 0.3095 | 17.5512 |  |
| MUTYH | -0.0348 | 0.6212 | 15.0585 |  |
| NF1 | -0.0844 | 0.2298 | 14.0124 |  |
| NTRK3 | -0.0140 | 0.8428 | 13.8522 |  |
| NUMA1 | -0.0489 | 0.4877 | 16.7333 |  |
| PAX5 | -0.0796 | 0.2576 | 13.2127 |  |
| PGR | -0.0248 | 0.7245 | 14.3941 |  |
| PHF6 | -0.0058 | 0.9346 | 13.4835 |  |
| PMS1 | -0.0219 | 0.7559 | 15.2495 |  |
| PRDM1 | -0.0004 | 0.9959 | 14.3366 |  |
| PRDM2 | -0.0488 | 0.4879 | 14.1393 |  |
| PREX2 | -0.0313 | 0.6568 | 13.2312 |  |
| PRKAR1A | -0.0308 | 0.6624 | 16.5292 |  |
| PRKDC | -0.0809 | 0.2502 | 15.4145 |  |
| PTPRD | -0.0449 | 0.5235 | 14.4119 |  |
| RAB40AL | -0.0991 | 0.1583 | 13.2453 |  |
| RABEP1 | -0.0687 | 0.3290 | 17.3346 |  |
| RANBP17 | -0.0771 | 0.2728 | 13.2919 |  |
| RAP1GDS1 | -0.0263 | 0.7083 | 15.7314 |  |
| RASSF1 | -0.0445 | 0.5278 | 15.2786 |  |
| RBBP8 | -0.0025 | 0.9714 | 14.6984 |  |
| RBP1 | -0.1739 | 0.0128 | 16.9774 |  |
| RNASEL | -0.0623 | 0.3763 | 16.1561 |  |
| RPTOR | -0.0351 | 0.6183 | 14.5302 |  |
| RUNX1 | -0.0227 | 0.7477 | 15.3497 |  |
| RUNX1T1 | -0.0367 | 0.6023 | 14.0611 |  |
| SDHAF2 | -0.0895 | 0.2033 | 17.3818 |  |
| SDHB | -0.1287 | 0.0665 | 17.9386 |  |
| SETD2 | -0.0426 | 0.5454 | 17.2496 |  |
| SFRP2 | -0.0279 | 0.6922 | 18.4511 |  |
| SMAD2 | -0.0062 | 0.9302 | 14.1585 |  |
| SMAD3 | -0.0963 | 0.1708 | 16.4175 |  |
| SMAD4 | -0.0969 | 0.1679 | 17.3092 |  |
| SMARCA4 | -0.0297 | 0.6736 | 17.6729 |  |
| SOCS1 | -0.0933 | 0.1843 | 15.7624 |  |
| SOCS3 | -0.0454 | 0.5192 | 14.8089 |  |
| SPEN | -0.0853 | 0.2251 | 17.2409 |  |
| SRGAP3 | -0.0210 | 0.7651 | 14.4660 |  |
| STK11 | -0.0854 | 0.2244 | 15.3924 |  |
| TCEA1 | -0.0642 | 0.3619 | 15.5099 |  |
| TET1 | -0.0380 | 0.5899 | 14.1420 |  |
| TFAP2A | -0.0262 | 0.7096 | 15.2176 |  |
| THBS1 | -0.0850 | 0.2270 | 17.5950 |  |
| THRAP3 | -0.1031 | 0.1421 | 15.7921 |  |
| TIMP3 | -0.0010 | 0.9886 | 17.6612 |  |
| TLX3 | -0.0153 | 0.8277 | 13.4299 |  |
| TMEFF2 | -0.0201 | 0.7758 | 13.5501 |  |
| TMEM127 | -0.0346 | 0.6228 | 15.6207 |  |
| TP73 | -0.0772 | 0.2723 | 13.1015 |  |
| TRIM33 | -0.0358 | 0.6112 | 15.7066 |  |
| TSC1 | -0.0164 | 0.8158 | 15.6086 |  |
| TSC2 | -0.0982 | 0.1622 | 15.2237 |  |
| YWHAE | -0.0193 | 0.7836 | 15.5636 |  |
| ZNF331 | -0.0028 | 0.9683 | 14.5922 |  |
| ZNF668 | -0.0229 | 0.7448 | 16.0503 |  |

B)

| **Gene** | | **Estimate** | | **P value** | | **Mean Expression** | |
| --- | --- | --- | --- | --- | --- | --- | --- |
| ATRX | -0.0013 | | 0.9850 | | 13.9469 | |  |
| BLID | -0.1188 | | 0.0906 | | 13.9765 | |  |
| BLM | -0.0764 | | 0.2777 | | 14.6813 | |  |
| BMP7 | -0.0179 | | 0.7990 | | 14.3881 | |  |
| BRCA1 | -0.0176 | | 0.8027 | | 14.9580 | |  |
| BRCA2 | -0.0017 | | 0.9810 | | 13.4017 | |  |
| BRIP1 | -0.1504 | | 0.0318 | | 13.8642 | |  |
| BTG1 | -0.0686 | | 0.3295 | | 18.6391 | |  |
| BUB1B | -0.0014 | | 0.9841 | | 14.8483 | |  |
| CASC5 | -0.0248 | | 0.7244 | | 13.9560 | |  |
| CCNB1IP1 | -0.0924 | | 0.1886 | | 14.7104 | |  |
| CD2 | -0.0996 | | 0.1563 | | 16.4594 | |  |
| CDH1 | -0.0434 | | 0.5375 | | 18.1575 | |  |
| CDH11 | -0.0247 | | 0.7257 | | 17.7525 | |  |
| CDK12 | -0.1043 | | 0.1375 | | 16.3371 | |  |
| CDKN1A | -0.0502 | | 0.4759 | | 15.6141 | |  |
| CDKN1C | -0.0564 | | 0.4230 | | 16.0217 | |  |
| CDKN2A | -0.1455 | | 0.0379 | | 14.3086 | |  |
| CHD5 | -0.0255 | | 0.7168 | | 13.7651 | |  |
| CHD6 | -0.0342 | | 0.6275 | | 15.8235 | |  |
| CHEK1 | -0.0315 | | 0.6542 | | 15.1712 | |  |
| CHEK2 | -0.1116 | | 0.1120 | | 14.5868 | |  |
| CHFR | -0.0462 | | 0.5116 | | 17.0289 | |  |
| CHN1 | -0.1318 | | 0.0602 | | 14.8979 | |  |
| CIITA | -0.1004 | | 0.1530 | | 14.5010 | |  |
| CLTCL1 | -0.0321 | | 0.6484 | | 14.1098 | |  |
| CNBP | -0.1700 | | 0.0151 | | 16.3166 | |  |
| COX6C | -0.0124 | | 0.8605 | | 19.2678 | |  |
| CTNNB1 | -0.0667 | | 0.3435 | | 15.9912 | |  |
| DAPK1 | -0.1735 | | 0.0131 | | 15.6762 | |  |
| DDIT3 | -0.0390 | | 0.5795 | | 16.0630 | |  |
| DKK1 | -0.0305 | | 0.6653 | | 14.3922 | |  |
| ELAC2 | -0.0832 | | 0.2367 | | 16.1477 | |  |
| EMP3 | -0.0929 | | 0.1864 | | 17.4996 | |  |
| EPHA5 | -0.0245 | | 0.7284 | | 13.2599 | |  |
| EPHA6 | -0.0975 | | 0.1653 | | 13.3497 | |  |
| ERCC2 | -0.0053 | | 0.9396 | | 15.5522 | |  |
| ERCC3 | -0.0620 | | 0.3782 | | 15.5411 | |  |
| EXT2 | -0.0684 | | 0.3307 | | 15.4237 | |  |
| FAM46C | -0.0795 | | 0.2583 | | 16.9997 | |  |
| FANCA | -0.0477 | | 0.4977 | | 13.7107 | |  |
| FANCC | -0.0026 | | 0.9707 | | 14.0886 | |  |
| FANCE | -0.0240 | | 0.7338 | | 15.8101 | |  |
| FANCI | -0.0836 | | 0.2344 | | 15.5721 | |  |
| FANCL | -0.1365 | | 0.0515 | | 14.9136 | |  |
| FAS | -0.0390 | | 0.5793 | | 14.8132 | |  |
| FAT1 | -0.0256 | | 0.7162 | | 17.3311 | |  |
| FBXW7 | -0.0699 | | 0.3205 | | 14.7353 | |  |
| FHIT | -0.1178 | | 0.0932 | | 14.5957 | |  |
| FOXL2 | -0.1411 | | 0.0441 | | 13.3440 | |  |
| FOXO1 | -0.0258 | | 0.7146 | | 16.4032 | |  |
| GATA5 | -0.1373 | | 0.0502 | | 13.5412 | |  |
| GMPS | -0.0775 | | 0.2704 | | 17.0642 | |  |
| GSTP1 | -0.0014 | | 0.9842 | | 18.4426 | |  |
| HERPUD1 | -0.0438 | | 0.5337 | | 17.6315 | |  |
| IGFBP3 | -0.0180 | | 0.7983 | | 17.3472 | |  |
| IKZF1 | -0.0348 | | 0.6209 | | 14.8119 | |  |
| IL21R | -0.1125 | | 0.1092 | | 14.0628 | |  |
| LMNA | -0.0583 | | 0.4075 | | 17.5236 | |  |
| MAL | -0.0373 | | 0.5964 | | 14.6452 | |  |
| MLF1 | -0.0316 | | 0.6541 | | 14.8246 | |  |
| MLH1 | -0.0214 | | 0.7617 | | 16.5589 | |  |
| MLLT11 | -0.0240 | | 0.7332 | | 16.2646 | |  |
| MSH2 | -0.1006 | | 0.1521 | | 14.7562 | |  |
| MSH6 | -0.0823 | | 0.2420 | | 17.5512 | |  |
| MTUS2 | -0.0431 | | 0.5402 | | 13.2167 | |  |
| MUTYH | -0.0187 | | 0.7906 | | 15.0585 | |  |
| NF2 | -0.0952 | | 0.1756 | | 13.5838 | |  |
| PALB2 | -0.0779 | | 0.2681 | | 15.3981 | |  |
| PAX5 | -0.0889 | | 0.2063 | | 13.2127 | |  |
| PHF6 | -0.0703 | | 0.3176 | | 13.4835 | |  |
| PLAG1 | -0.0161 | | 0.8191 | | 13.6951 | |  |
| PML | -0.0076 | | 0.9144 | | 13.8870 | |  |
| PMS1 | -0.0374 | | 0.5955 | | 15.2495 | |  |
| PRDM1 | -0.0945 | | 0.1790 | | 14.3366 | |  |
| PREX2 | -0.0556 | | 0.4293 | | 13.2312 | |  |
| PRKAR1A | -0.0942 | | 0.1804 | | 16.5292 | |  |
| PRKDC | -0.0798 | | 0.2563 | | 15.4145 | |  |
| PRLR | -0.0225 | | 0.7497 | | 14.7949 | |  |
| PTPRD | -0.0128 | | 0.8563 | | 14.4119 | |  |
| RAB40AL | -0.1972 | | 0.0047 | | 13.2453 | |  |
| RAD51B | -0.0780 | | 0.2675 | | 13.9763 | |  |
| RAD51C | -0.1491 | | 0.0333 | | 15.4363 | |  |
| RAD51D | -0.0693 | | 0.3245 | | 14.4466 | |  |
| RANBP17 | -0.0678 | | 0.3356 | | 13.2919 | |  |
| RASSF5 | -0.0508 | | 0.4708 | | 14.5639 | |  |
| RBBP8 | -0.0517 | | 0.4629 | | 14.6984 | |  |
| RBP1 | -0.1176 | | 0.0940 | | 16.9774 | |  |
| RMI2 | -0.0087 | | 0.9022 | | 16.1039 | |  |
| RPTOR | -0.0373 | | 0.5968 | | 14.5302 | |  |
| RUNX3 | -0.0945 | | 0.1786 | | 15.1754 | |  |
| SDHAF2 | -0.2143 | | 0.0021 | | 17.3818 | |  |
| SDHB | -0.1346 | | 0.0550 | | 17.9386 | |  |
| SLX4 | -0.0092 | | 0.8961 | | 15.8230 | |  |
| SMAD2 | -0.0204 | | 0.7720 | | 14.1585 | |  |
| SMARCA4 | -0.0266 | | 0.7057 | | 17.6729 | |  |
| SOCS1 | -0.0468 | | 0.5064 | | 15.7624 | |  |
| SPECC1 | -0.0448 | | 0.5249 | | 14.1781 | |  |
| SRGAP3 | -0.0357 | | 0.6122 | | 14.4660 | |  |
| STK11 | -0.0150 | | 0.8319 | | 15.3924 | |  |
| SYK | -0.0274 | | 0.6969 | | 16.3501 | |  |
| TCEA1 | -0.0029 | | 0.9671 | | 15.5099 | |  |
| TET1 | -0.0135 | | 0.8485 | | 14.1420 | |  |
| TFAP2A | -0.0213 | | 0.7627 | | 15.2176 | |  |
| THRAP3 | -0.0139 | | 0.8436 | | 15.7921 | |  |
| TMEFF2 | -0.0639 | | 0.3639 | | 13.5501 | |  |
| TMEM127 | -0.0150 | | 0.8318 | | 15.6207 | |  |
| TNFAIP3 | -0.0886 | | 0.2078 | | 16.0023 | |  |
| TOP2A | -0.0328 | | 0.6418 | | 16.9282 | |  |
| TP73 | -0.0708 | | 0.3145 | | 13.1015 | |  |
| TUBB3 | -0.0276 | | 0.6949 | | 16.2461 | |  |
| VDR | -0.0587 | | 0.4045 | | 14.2406 | |  |
| XPA | -0.0031 | | 0.9653 | | 15.5822 | |  |
| ZNF668 | -0.0059 | | 0.9328 | | 16.0503 | |  |
| ZRSR2 | -0.0079 | | 0.9110 | | 16.0608 | |  |
