# Supplementary material for: The novel microRNAs hsa-miR-nov7 and hsa-miR-nov3 are over-expressed in locally advanced breast cancer
Source: PLoS One. 2020 Apr 16;15(4):e0225357. doi: 10.1371/journal.pone.0225357 (PMC7162276; doi:10.1371/journal.pone.0225357)
Supplement: S1 File — (DOCX) [file pone.0225357.s010.docx]

**miRDeep algorithm**

This algorithm exploits Dicer’s miRNA precursor processing along with integrating massively parallel sequencing data into a simple probabilistic model. First it searches for potential precursor secondary structure and reads corresponding to them. It makes sure that precursor sequence has reads aligning to mature, star and loop of precursor, by-products of Dicer processing. Algorithm searches for both structural and miRNA signatures and scores precursor sequences based on of energetically stable it is, conservation on phylogenetic distance, number of reads mapping to it as wells number of reads aligning to all three Dicer products. The algorithm rejects reads that align to multiple positions (>5) in the genome. It also does not consider reads that map in to already annotated non - coding RNA regions in the genome. Rest of the reads are assessed both structural stability and closeness to miRNA signatures. The precursor sequence is assigned mature position based on where majority of the reads align followed potential star sequence with a base pairing with an overhang that is phylogenetically conserved typically of lengths 2 – 3 nts. Stem loop sequence with at least 14 nts between mature and star sequence that can form an unbifurcated hairpin structure is defined. Finally miRDeep2 give probability score for each predicted sequence, ruling out the possibility of background hairpin formation.

[1, 2].

1. Friedlander MR, Chen W, Adamidi C, Maaskola J, Einspanier R, Knespel S, et al. Discovering microRNAs from deep sequencing data using miRDeep. Nat Biotechnol. 2008;26(4):407-15. Epub 2008/04/09. doi: 10.1038/nbt1394. PubMed PMID: 18392026.

2. Friedländer MR, Mackowiak SD, Li N, Chen W, Rajewsky N. miRDeep2 accurately identifies known and hundreds of novel microRNA genes in seven animal clades. Nucleic Acids Res. 2012;40(1):37-52. doi: 10.1093/nar/gkr688. PubMed PMID: 21911355; PubMed Central PMCID: PMCPMC3245920.
